# Supplementary material for: Domain adaptation for supervised integration of scRNA-seq data
Source: Commun Biol. 2023 Mar 16;6:274. doi: 10.1038/s42003-023-04668-7 (PMC10020569; doi:10.1038/s42003-023-04668-7)
Supplement: Supplementary file 2 — Supplementary Information [file 42003_2023_4668_MOESM2_ESM.pdf]

Supplementary Information for

"Domain adaptation for supervised integration of scRNA-seq data"

Yutong Sun[1] and Peng Qiu[2,*]

[1]School of Electrical and Computer Engineering, Georgia Institute of Technology,

[2]Department of Biomedical Engineering, Georgia Institute of Technology and Emory University

Supplementary Note 1: Detail references of datasets included in the data collections analyzed in this study

In this study, we analyzed 5 data collections, each containing multiple datasets that can be integrated. Some of the data collections were obtained from previous benchmarking studies, while others were assembled by datasets published in separate papers. In Table S1, we provide detailed references of every individual dataset included in the data collections analyzed in this study.

Supplementary Table 1. Detailed references of datasets included in the 5 data collections (pancreas, PBMC, gut, pancreatic islet, HSCs)

Data collection	batch	reference
pancreas	Baron	Baron M, Veres A, Wolock S L, et al. A single-cell transcriptomic map of the human and mouse pancreas reveals inter-and intra-cell population structure[J]. Cell systems, 2016, 3(4): 346-360. e4.
	Muraro	Muraro MJ, Dharmadhikari G, Grun D, Groen N, Dielen T, Jansen E, et al. A single-cell transcriptome atlas of the human pancreas. Cell Syst. 2016;3:385–394.e3.
	Segerstolpe	Segerstolpe A, Palasantza A, Eliasson P, Andersson E-M, Andreasson A-C, Sun X, et al. Single-cell transcriptome profiling of human pancreatic islets in health and type 2 diabetes. Cell Metab. 2016;24:593–607.
	Wang	Wang YJ, Schug J, Won K-J, Liu C, Naji A, Avrahami D, et al. Single-cell transcriptomics of the human endocrine pancreas. Diabetes. 2016;65:3028–38.
	Xin	Xin Y, Kim J, Okamoto H, Ni M, Wei Y, Adler C, et al. RNA sequencing of single human islet cells reveals type 2 diabetes genes. Cell Metab. 2016;24:608–15.
PBMC	control	Butler A, Hoffman P, Smibert P, et al. Integrating single-cell transcriptomic data across different conditions, technologies, and species[J]. Nature biotechnology, 2018, 36(5): 411-420.
	stim	
	PBMC3k	Satija Lab (2020). pbmc3k.SeuratData: 3k PBMCs from 10X Genomics. R package version 3.1.4.
	10x 3'	Zheng GXY, Terry JM, Belgrader P, Ryvkin P, Bent ZW, Wilson R, et al. Massively parallel digital transcriptional profiling of single cells. Nat Commun. 2017;8:14049.
	10x 5'	
Gut	Bigaeva	Bigaeva, E., Uniken Venema, W. T., Weersma, R. K. & Festen, E. A. Understanding human gut diseases at single-cell resolution. Hum. Mol. Genet. 29, R51–R58 (2020).
	Huang	Huang, B. et al. Mucosal profiling of pediatric-onset colitis and ibd reveals common pathogenics and therapeutic pathways. Cell 179, 1160–1176 (2019).
	Parikh	Parikh, K. et al. Colonic epithelial cell diversity in health and inflammatory bowel disease. Nature 567, 49–55 (2019).
	Wang	Wang, Y. et al. Single-cell transcriptome analysis reveals differential nutrient absorption functions in human intestine. J. Exp. Medicine 217 (2020).
pancreatic islet	CEL-Seq	Muraro M J, Dharmadhikari G, Grün D, et al. A single-cell transcriptome atlas of the human pancreas[J]. Cell systems, 2016, 3(4): 385-394. e3.
	CEL-Seq2	
	Fluidigm C1	
	Smart-Seq2	
HSCs	old	Kowalczyk M S, Tirosh I, Heckl D, et al. Single-cell RNA-seq reveals changes in cell cycle and differentiation programs upon aging of hematopoietic stem cells[J]. Genome research, 2015, 25(12): 1860-1872.
	young	

Supplementary Note 2: Comparison of computational efficiency

We examined and compared the computational efficiency of SIDA, four unsupervised algorithms (Seurat, Harmony, Limma, scAlign), and two supervised algorithms (scAlign+ and LAmbDA) across three data collections (pancreas, PBMC and gut). In our implementation of the integration algorithms that rely on deep learning and GPU, the GPU we used was NVIDIA Quadro P2200.

Table S2 shows that algorithms without deep learning strategy (Seurat, Harmony and Limma) are computationally much cheaper than the other four deep-learning-based algorithms (SIDA, scAlign, scAlign+, and LAmbDA). Among all the algorithms, SIDA achieves best integration performance and has the longest computing time. This result represents a trade-off between performance and computational cost. In terms of how to decide which integration method should be used, we would suggest the following. When integrating newly generated datasets without prior analysis and cell type labels, we have to use unsupervised integration algorithms, such as Seurat and Harmony. When integrating previously analyzed datasets with cell type labels available, we believe it is better to use supervised integration, and we would prefer our method, SIDA. Although Table S2 shows that SIDA requires longer computing time compared to other deep-learning-based supervised integration algorithms, we have demonstrated in Table 1 and Figure 3c that SIDA significantly outperformed the other methods in the gut data collection, which is the most challenging data collection analyzed in this study.

Supplementary Table 2. Comparison of computation efficiency of SIDA, four unsupervised algorithms (Seurat, Harmony, Limma, scAlign), and two supervised algorithms (scAlign+ and LAmbDA) applied to three data collections.

		**SIDA**	**Seurat**	**Harmony**	**Limma**	**scAlign**	**scAlign+**	**LAmbDA**
**Pancreas**	**Computing time**	8 hours	9 minutes	49 seconds	1 minute	7.5 hours	6 hours	9 hours
	**Memory consuming**	2632MB RAM +3865MBGPU	11562.7MB RAM	13949.9MB RAM	13433.6MB RAM	10024.1MB RAM	10011.7MB RAM	954MB RAM +3875MB
**PBMC**	**Computing time**	28 hours	32 minutes	2 minutes	3 minutes	6 hours	5 hours	12 hours
	**Memory consuming**	797.9MB RAM +4149MBGPU	29454.4MB RAM	23893.3MB RAM	32903.1MB RAM	20457MB RAM	27870MB RAM	983.7MB RAM +3822MB
**Gut**	**Computing time**	25 hours	20 minutes	88 seconds	75 seconds	2 hours	3.3 hours	11 hours
	**Memory consuming**	1098.9MB RAM +3945MBGPU	21552.6MB RAM	17746.2MB RAM	17105.5MB RAM	47004.7MB RAM	47063.4MB RAM	1243.4MB RAM +3862MB

Supplementary Note 3: UMAP visualization of integrated data

In Figure 2 of the main text, we provided tSNE visualizations of the embedding space generated by SIDA, four unsupervised algorithms (Seurat, Harmony, Limma, scAlign) and two supervised algorithms (scAlign+ and LAmbDA), across three data collections (pancreas, PBMC and gut). In Figures 4 and 5 of the main text, we provided tSNE visualizations of the embedding space generated by SIDA, scAlign and scAlign+ based on two data collections (pancreatic islet and HSC). In this supplemental section, we provide the corresponding UMAP visualizations of these analyses, in Figures S1, S2 and S3, respectively.

For the pancreas data collection, the integration results are shown in the UMAP visualizations in Figure S1(a-b), colored by cell types and batch labels. Seurat and Harmony successfully mixed the different batches, as shown in the 2nd and 3rd columns in Figure S1(b). However, when colored by cell type labels, the 2nd and 3rd columns of Figure S1(a) show that Seurat and Harmony improperly aligned some of the distinct cell types in different batches, e.g. stellate and mesenchymal, acinar and ductal. In the 4th – 6th columns of Figure S1(a-b), we observe that Limma, scAlign and scAlign+ performed poorly, where the same cell type in different batches did not align and mix with each other. The last column of Figure S1(a) shows that LAmbDA successfully aggregated and mixed the different batches. However, the last column in Figure S1(b) shows that LAmbDA failed to separate different cell types properly. From the 1st column in Figure S1(a-b), we can observe that SIDA achieved better cell type separation and batch mixing, compared to the four unsupervised and the two supervised methods.

For the PBMC data collection, the integration results are shown in the UMAP visualization in Figure S1(c-d), colored by cell types and batch labels. The 1st column of Figure S1(c-d) shows that SIDA performed well on PBMC data collection, achieving proper mixing of different batches. Based on the 2nd and 3rd columns of Figure S1(c-d), Seurat and Harmony mixed the different batches, but improperly aligned two similar cell types: CD4 T and CD8 T. Based on the 4th - 6th columns of Figure S1(c-d), we observe that Limma, scAlign and scAlign+ failed to properly integrate the PBMC data collection, which is consistent with their performance in the pancreas data collection. From the last column of Figure S1(c-d), we can observe that LAmbDA did not separate different cell types properly.

For the gut data collection, the integration results are shown in the UMAP visualization in Figure S1(e-f), colored by cell types and batch labels. As shown in the 2nd – 6th columns of Figure S1(e-f), Seurat, Harmony Limma, scAlign and scAlign+ did not effectively mix the batches, and did not properly align corresponding cell types in different batches. The last column of Figure S1(e-f) shows that LAmbDA successfully mixed the four different batches, but improperly aligned different cell types.

These observations and interpretations based on the UMAP visualizations are consistent with the interpretations of the tSNE visualization in the main text.

Supplementary Figure 1. UMAP visualization of SIDA, four unsupervised algorithms (Seurat, Harmony, Limma, scAlign), and two supervised algorithms (scAlign+ and LAmbDA) applied to three data collections.

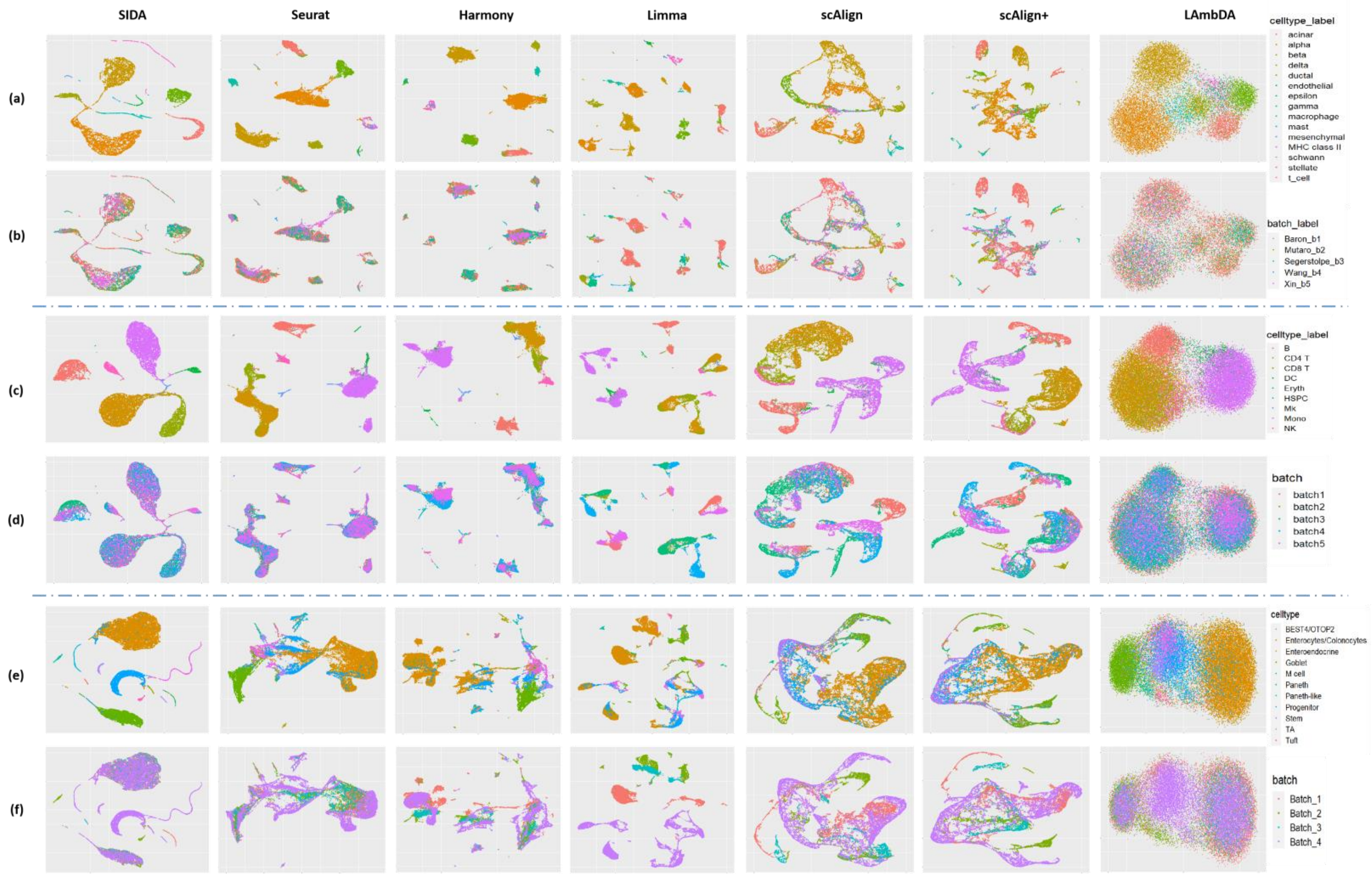

(a-b) integration of pancreas data collection colored by cell types and batch labels; **(c-d)** integration of PBMC data collection colored by cell types and batch labels; **(e-f)** integration of gut data collection colored by cell types and batch labels

Supplementary Figure 2. UMAP visualization of SIDA, scAlign+ and scAlign applied to HSCs datasets.

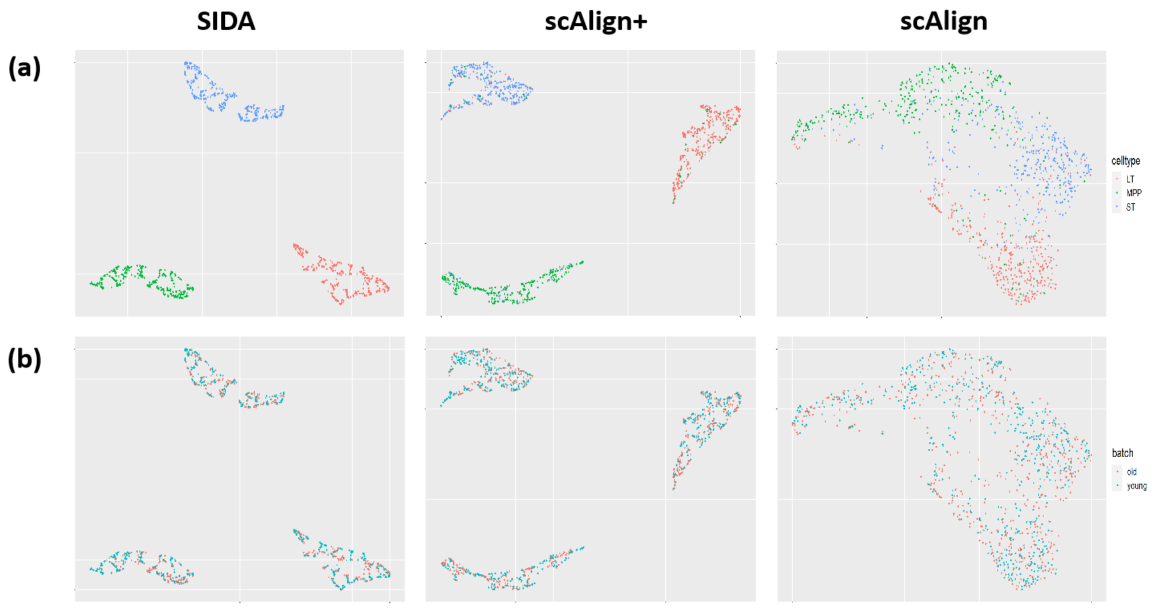

(a) integration colored by cell types; **(b)** integration colored by batch labels.

Supplementary Figure 3. UMAP visualization of SIDA, scAlign+ and scAlign applied to pancreatic islet datasets.

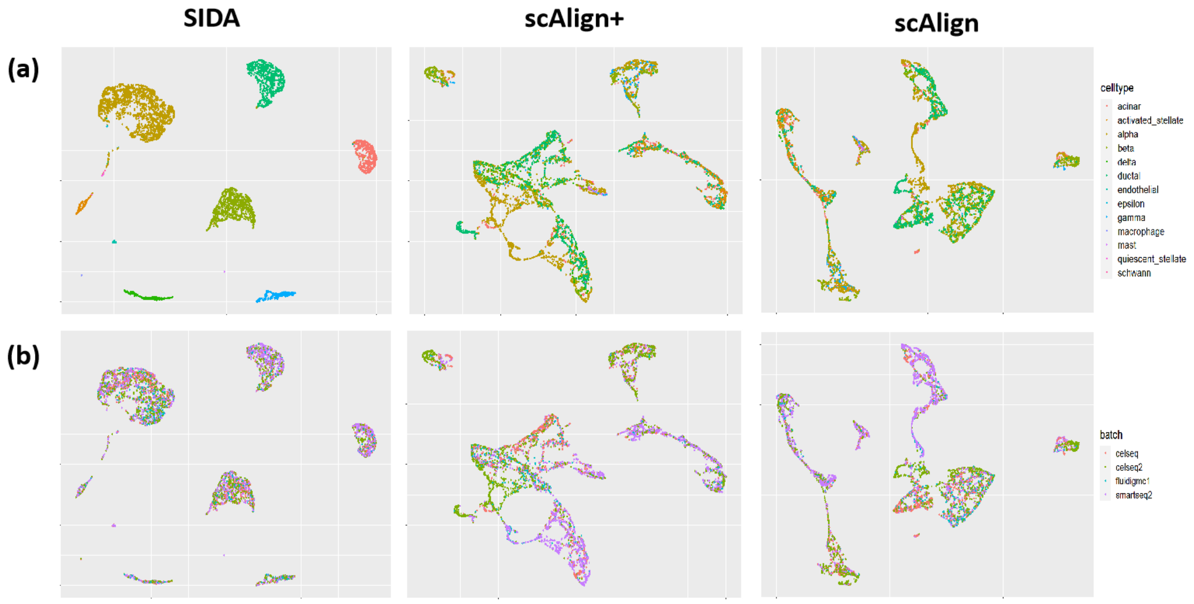

(a) integration colored by cell types; **(b)** integration colored by batch labels.

Supplementary Note 4: Evaluation of robustness and reproducibility of SIDA

As described in the Methods section of the main text, training the SIDA model requires sampling of a subset of cells (e.g., 400) from each cell type and each batch, and these subsets of cells are used to form training pairs for the SIDA model to learn the differences of corresponding cell types across different batches. Given the stochasticity involved in random sampling, it is important to evaluate the robustness and reproducibility of SIDA with respect to the random sampling. Therefore, we performed a consistency evaluation experiment on the pancreas, PBMC and gut data collections. For each data collection, we trained three SIDA models using cells sampled with different random seeds. We used the 6 metrics (true positive rate, positive rate, kBET, LISI, ASW, ARI) and tSNE visualization to examine the trained models. If the metrics are similar across the SIDA models constructed from the same data collection, we can be more confident in the robustness and reproducibility of SIDA.

The evaluations of the 6 metrics across the three data collections are summarized in Figure S4, where each panel includes the evaluation on one data collection. The three colors represent the three models trained based on different random seeds. For majority of the metrics in the three data collections (each group of bars with the three colors), SIDA models achieved low variation, which was much lower than the variations among different data collections, especially for the metrics dominated by cell type purity. Therefore, this result demonstrated SIDA's robustness and reproducibility.

Figure S5 shows the tSNE visualization of the embedding space generated by the three SIDA models trained by randomly sampled cells from the three data collections. In each section of Figure S5, we can observe that all the three SIDA models achieve highly similar tSNE visualization of the embedding space, except for some rotations and rearrangements of clusters which tSNE tends to do. Therefore, consistency of tSNE visualizations of the embedding space further supported SIDA's robustness and reproducibility.

Supplementary Figure 4. Comparing three SIDA models trained by samples achieved with different random seeds using 6 quantitative evaluation metrics for batch mixing and cell type separation.

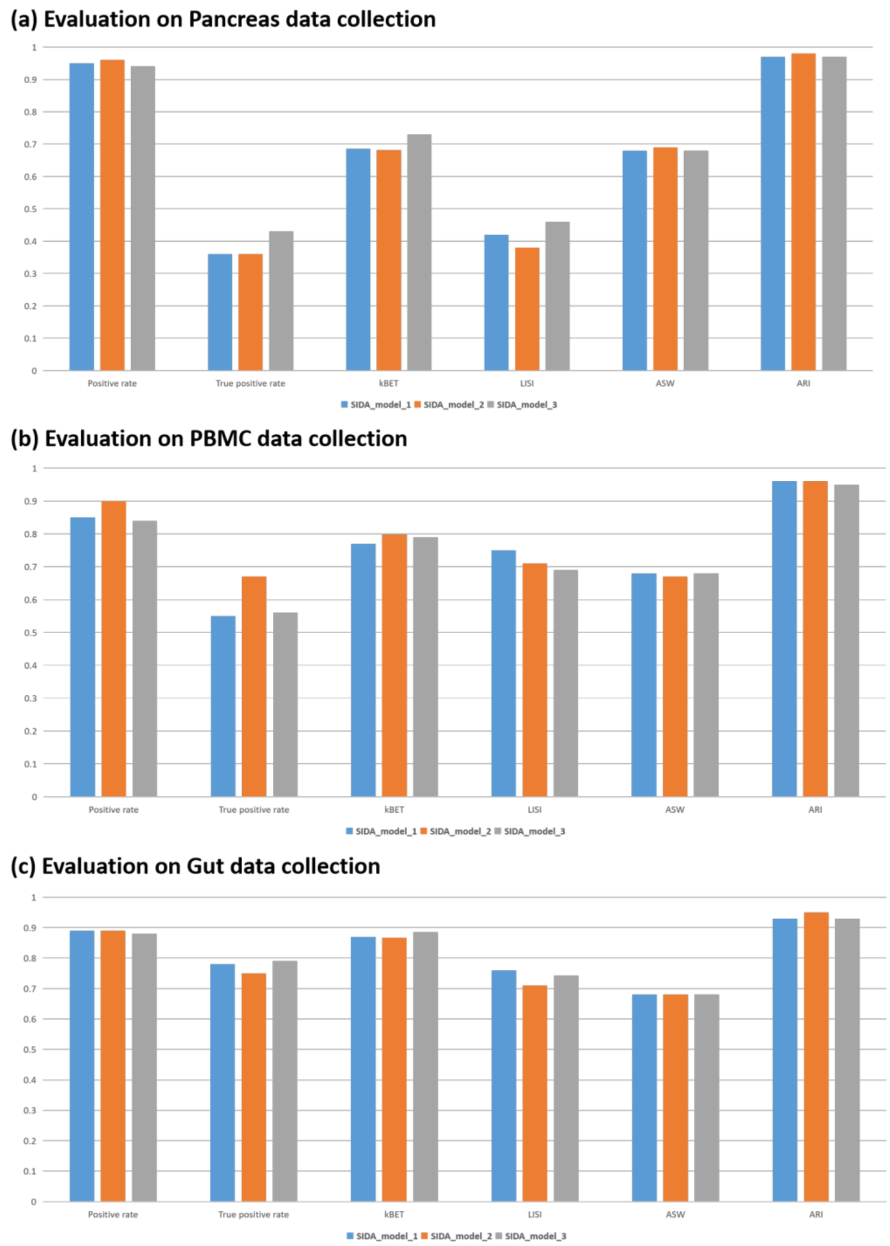

(a) Evaluation based on the pancreas data collections; **(b)** Evaluation based on the PBMC data collection; **(c)** Evaluation based on the gut data collection.

Supplementary Figure 5. tSNE visualization of three SIDA models trained by samples achieved with different random seeds applied to three data collections.

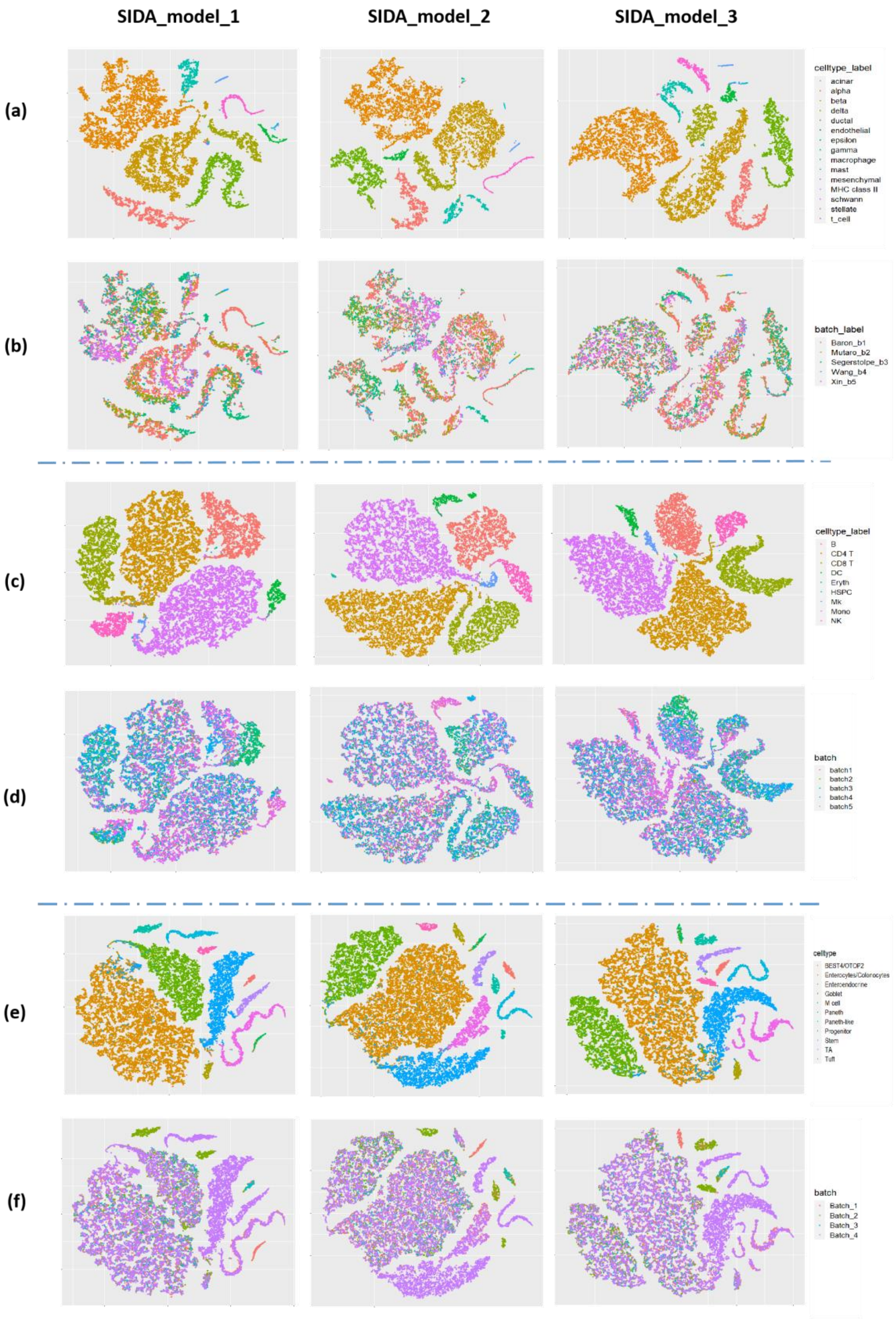

(a-b) integration of pancreas data collection colored by cell types and batch labels; **(c-d)** integration of PBMC data collection colored by cell types and batch labels; **(e-f)** integration of gut data collection colored by cell types and batch labels
